# Gummy stem blight: One disease, three pathogens

**DOI:** 10.1111/mpp.13339

**Published:** 2023-05-02

**Authors:** Rewa Seblani, Anthony P. Keinath, Gary Munkvold

**Affiliations:** ^1^ Plant Pathology, Entomology, and Microbiology Iowa State University Ames Iowa USA; ^2^ Coastal REC, Clemson University Charleston South Carolina USA

**Keywords:** *Didymella bryoniae*, gummy stem blight, *Stagonosporopsis caricae*, *Stagonosporopsis citrulli*, *Stagonosporopsis cucurbitacearum*

## Abstract

Gummy stem blight (GSB) is a major disease of cucurbits worldwide. It is caused by three fungal species that are morphologically identical and have overlapping geographic and host ranges. Controlling GSB is challenging due to the lack of resistant cultivars and the pathogens' significant ability to develop resistance to systemic fungicides. The causal agent of GSB is recognized as a complex of three phylogenetically distinct species belonging to domain Eukaryota, kingdom Fungi, phylum Ascomycota, subphylum Pezizomycotina, class Dothideomycetes, subclass Pleosporomycetida, order Pleosporales, family Didymellaceae, genus *Stagonosporopsis*, species *cucurbitacearum*, *citrulli*, and *caricae*. Pycnidia are tan with dark rings of cells around the ostiole measuring 120–180 μm in diameter. Conidia are 6–13 μm long, hyaline, cylindrical with round ends, and non‐ or monoseptate. Pseudothecia are black and globose in shape and have a diameter of 125–213 μm. Ascospores are 14–18 × 4–6 μm long, hyaline, ellipsoidal with round ends, and monoseptate with a distinct constriction at the septum. Eight ascospores are found per ascus. The upper end of the apical cell is pointed, whereas the lower end of the bottom cell is blunt. Species‐specific PCR primers that can be used in a multiplex conventional PCR assay are available. The GSB species complex is pathogenic to 37 species of cucurbits from 21 different genera. *S. cucurbitacearum* and *S. citrulli* are specific to cucurbits, while *S. caricae* is also pathogenic to papaya and babaco‐mirim (*Vasconcellea monoica*), a related fruit. Under favourable environmental conditions, symptoms can appear 3–12 days after spore germination. Leaf spots often start at the leaf margin or extend to the margins. Spots expand and coalesce, resulting in leaf blighting. Active lesions are typically water‐soaked. Cankers are observed on crowns, main stems, and vines. Red to amber gummy exudates are often seen on the stems after cankers develop on cortical tissue.

## Graphical abstract

Gummy stem blight is an important disease of many cucurbit crops worldwide; the causal agents are three species of *Stagonosporopsis* with identical morphology and overlapping host ranges.

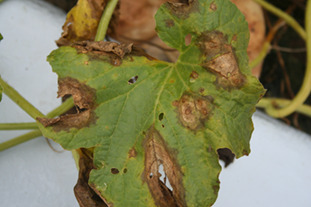

## 1 INTRODUCTION

Cucurbits, members of the family Cucurbitaceae, are important economic crops worldwide. In the United States, annual field production is around 109 million tonnes on 229,000 ha, with a value of $1.43 billion. Most of the cucurbit production is concentrated in Florida, Michigan, California, Texas, Georgia, and North Carolina (Cantliffe et al., 2007). In 2019, watermelon production totalled 44.4 t/ha, while cantaloupe, cucumber, and squash totalled 35, 17.3, and 19.6 t/ha, respectively (USDA‐NASS, 2020). In 2020, global watermelon production was 101.6 million tonnes, with China producing approximately 60% of the total. Turkey, Iran, and Brazil ranked second to fourth, each having an annual production of 2–3 million tonnes in 2020 (FAO, 2021). Similarly, global cucumber production was approximately 91.3 million tonnes in 2020, with China producing about 72.8 million tonnes, followed distantly by Turkey, Russia, and Iran. For melons, global production was about 28.5 million tonnes; China produced nearly half of the total (13.8 million tonnes), with Turkey, India, and Iran producing <2 million tonnes each. In 2020, approximately 28 million tonnes of squash and pumpkins were produced globally; leading producers were China (7.4 million tonnes) and India (5.1 million tonnes), followed by Ukraine, the Russian Federation, and the United States, each with slightly more than 1 million tonnes (FAO, 2021).

Cucurbits are affected by numerous plant‐pathogenic microorganisms, including fungi, oomycetes, bacteria, phytoplasmas, viruses, viroids, and nematodes (Keinath et al., 2017). Many of the most important diseases are caused by fungi, resulting in significant yield and quality losses. Gummy stem blight (GSB), a ubiquitous fungal disease, affects all commonly cultivated cucurbits, especially in humid, temperate, semitropical, and tropical regions (Garampalli et al., 2016; Keinath, 2017). GSB affects cucurbit production globally, with significant impacts on profit and food security. GSB can decrease yield and reduce fruit quality (Keinath, 2000). Losses due to GSB can vary between 17% and 43% (Keinath, 2001; Keinath & Duthie, 1998).

GSB was first reported in 1823 by E. Fries on an unknown cucurbit in Sweden. In 1869 it was found on bryony in Germany, and in 1885 it was found by G. Passerini on *Cucumis melo* in Italy. In 1891, GSB was reported by Fautrey and Roumeguere in France on cucumber and in Delaware on watermelon (Chester, 1891; Chiu & Walker, 1949). Furthermore, GSB was reported in 1917 in Florida on watermelon (Sherbakoff, 1917), where it is still considered a limiting factor in the watermelon industry. GSB is considered a potential risk in the global movement of plant pathogens as it can be on or in seeds and transplants, hence facilitating its movement from continent to continent (Keinath, 2011). By the 1980s, it had been reported in at least 70 countries on six different continents (CABI, 2021). Between 1993 and 2009, GSB was detected 16 times on melon and watermelon transplants in South Carolina (Keinath, 2009). It is currently found on every continent where cucurbits are grown, on 37 species of cucurbits from 21 different genera (Rennberger & Keinath, 2018).

International seed trade increases the risk of seedborne pathogen dissemination (Walcott, 2008). The pathogen can be present in and on the seed coat of cucurbits including the periplasm and in the tissue of the cotyledons (Lee et al., 1984). Two early reports of GSB in 1891 were believed to be from seedborne inoculum (Keinath, 2011). Brown et al. (1970) found three glasshouses with GSB using cucumber seeds from one source. Using a blotter test, the same seeds were germinated and 6% of seedlings (9/150) were diseased. Additionally, seedling infection was traced to contaminated seeds in pumpkin and cucumber (Lee et al., 1984). With the absence of symptoms on affected fruits, seeds of these symptomless fruits might be extracted for use (Keinath, 2011). Sowing infected seeds can reduce emergence, vigour, and yield. Even with low levels of seedborne inoculum, favourable epidemiological factors can result in high levels of disease in field or indoor plantings (Keinath, 1996a, 1996b; Neergard, 1977). Sudisha et al. (2006) demonstrated that *Stagonosporopsis* (species not determined) can be transmitted from seed to plant and vice versa.

Grafting of cucurbits is used in several countries to protect against soilborne pathogens (Davis et al., 2008). Crino et al. (2007) determined the effectiveness of eight commercial rootstocks (*Cucurbita maxima* × *Cucurbita moschata* and *Cucumis melo* genotypes) for their resistance to GSB. The inodorus F_1_ hybrid Incas was grafted onto each of the rootstocks and evaluated under greenhouse conditions. *Cucurbita* rootstocks RS 841, P 360, ES 99–13, and Elsi (*C. maxima* × *C. moschata*) were highly resistant to GSB fungi, showing almost no crown lesions and a low leaf disease index. Moreover, RS 841 grafted with Incas was reported as the best genotype that can improve productivity without having a negative effect on fruit quality (Crino et al., 2007).

Grafted cucurbits also can serve as a source of GSB for field‐grown crops. Regardless of the grafting method used, both rootstock and scion are wounded. The wounds may promote GSB development especially because the grafted plants are kept at high humidity or under frequent mist to promote graft union healing (Keinath & DuBose, 2017). In Tunisia, the first report of GSB was on grafted watermelon (Boughalleb et al., 2007).

## 2 TAXONOMY

Various names have been assigned to the causal agent(s) of GSB. In 1869, Bernhard Auerswald and Karl W. G. L. Fuckel, working independently, coined the same species name “*bryoniae*” but assigned the first GSB causal agent described to different genera (*Sphaerella* and *Sphaeria*, respectively). However, because Auerswald published his report a year earlier, he was given priority. In 1880, Pier Andrea Saccardo created the genus *Didymella*. Heinrich Rehm named the causal agent *Didymella bryoniae* in 1881. Previously, the teleomorph of this fungus was known as *Didymella bryoniae* (Auersw.) Rehm (=*Mycosphaerella citrullina* and *Mycosphaerella melonis*), while its anamorph was *Phoma cucurbitacearum* (Fr.:Fr.) Sacc. (=*Ascochyta cucumis*).

Other *Phoma* species have been isolated from plants exhibiting GSB symptoms. However, some *Phoma* species did not produce GSB symptoms on inoculated plants (Keinath et al., 1995). To better understand the molecular and phylogenetic relationship of *D. bryoniae* and *Phoma*, Somai et al. (2002) used random amplified polymorphic DNA (RAPD) fingerprinting to group 59 isolates of *D. bryoniae* and *Phoma* into four phylogenetic groups denoted as RAPD Group (RG) I, RG II, RG III, and RG IV. *D. bryoniae* isolates clustered in RG I, RG II, and RG IV, whereas *Phoma* isolates clustered in RG III. *D. bryoniae* isolates in RG I, RG II, and RG IV were indistinguishable based on pycnidia and pseudothecia production, shape and septation of conidia, and colony morphology. Moreover, the assignment of isolates into the RAPD groups was supported by the estimation of evolutionary distances using phylogenetic analysis using parsimony.

Using ribosomal DNA internal transcribed spacer (ITS) sequence analysis on 35 representative isolates belonging to the four RG groups, ITS‐4 and ITS‐5 primers successfully amplified a uniform fragment of approximately 500 bp. RG I and RG II isolates were found to have 99% sequence identity. Moreover, ITS sequence data showed a stronger relationship between RG I and RG II when compared to RAPD data. This can be linked to the ability of RAPD analysis to distinguish between closely related fungi. Additionally, the full genome is more subject to change than conserved regions such as the ITS.

In 2003, amplified fragment length polymorphism (AFLP) was used to further analyse the genetic variation among 102 isolates of *D. bryoniae* from 10 states and seven countries. Using cluster analysis, two groups and seven subgroups were delineated. Based on subsequent identifications, most RG I isolates were *Stagonosporopsis citrulli*, most RG II‐a isolates were *Stagonosporopsis cucurbitacearum*, and several RG II‐b isolates were *Stagonosporopsis caricae* (Stewart et al., 2015). Isolates from northern US states clustered separately from isolates from southern US states (Kothera et al., 2003). These results supported the hypothesis that GSB is composed of at least two genetically distinct groups.

In 2010, Aveskamp, Gryter, and Verkley used sequences from 28S nuclear ribosomal DNA (nrDNA), 18S nrDNA, 5.8S nrDNA, ITS regions 1 and 2, and part of the β‐tubulin gene region to analyse 159 taxa with affinities to *Phoma*. Most of the taxa were part of the family Didymellaceae and were segregated into 18 distinct clades with specific taxonomic characters. An isolate of *Phoma cucurbitacearum* was grouped into a new clade and was renamed *Stagonosporopsis cucurbitacearum* (Aveskamp et al., 2010). The changes to the International Code of Botanical Nomenclature in 2013 state that any fungus should have only one name, even if it has anamorph and teleomorph states (Taylor, 2011). The GSB causal agent became known as *S. cucurbitacearum*.

## 3 GSB SPECIES COMPLEX

The causal agent of GSB is now recognized as a complex of three phylogenetically distinct species that are all closely related and pathogenic to cucurbits. Using a multilocus sequencing approach of four loci (ITS, β‐tubulin [*BTUB*], chitin synthase I [*CHS*], and calmodulin [*CAL*]), Stewart et al. (2015) determined that fungi causing GSB comprised three phylogenetically distinct species: *S. cucurbitacearum* (Fr.:Fr.) Aveskamp, Gruyter & Verkley, *S. citrulli* M.T. Brewer & J.E. Stewart, and *S. caricae* (Sydow & P. Sydow) Aveskamp, Gruyter & Verkley. Because no previous name existed in the literature for the clade with isolates of *S. citrulli*, Stewart et al. (2015) named this species after the most common host represented in their collection, watermelon (*Citrullus lanatus*). For taxonomic clarity, it is important to note that *S. cucurbitacearum* is the “successor” taxon to the previous names *Didymella bryoniae* and *Phoma cucurbitacearum*, even though these names often inadvertently referred to isolates now named *S. citrulli*. In previous studies, the genetic groups RG II, RG I, and RG IV are now known to overlap with *S. cucurbitacearum*, *S. citrulli*, and *S. caricae*, respectively (Keinath et al., 1995; Somai et al., 2002; Stewart et al., 2015).

Aveskamp et al. (2010) reported the genetic relationship between *S. cucurbitacearum* and *S. caricae* as phylogenetically distinct sister species. Additionally, Stewart et al. (2015) demonstrated that *S. cucurbitacearum* and *S. citrulli* are sister species with an estimated divergence at 10,900 years before present (YBP). Moreover, *S. caricae* is the ancestral lineage with an estimated divergence from its sister species *S. cucurbitacearum* and *S*. *citrulli* at 72,900 YBP, which was before the domestication of papaya and cucurbits in the American tropics (Stewart et al., 2015). There are no morphological features that help differentiate between the three species.

Using draft genome sequences, Li et al. (2017) identified the mating type loci (*MAT1*) of three isolates for each of the three species *S. cucurbitacearum*, *S. caricae*, and *S. citrulli*. MAT1 was found to be structurally identical throughout the three species. Moreover, MAT1 in all three species contained both mating type genes, MAT1‐1‐1 and MAT1‐2‐1, required for sexual reproduction, which confirmed that the species are homothallic. Nonetheless, based on the number of amino acid substitutions detected, MAT1‐1‐1 and MAT1‐2‐1 were found to be more divergent in reproductive genes than in genes flanking MAT1. Additionally, positive selection was found for MAT1‐2‐1, most particularly in the sequence encoding the HMG‐box. This shows that even though the mating type genes are evolving rapidly in GSB fungi, differences in the mating system of the three species are not related to their divergence.

The GSB species complex is pathogenic to cucurbits; *S. caricae* is also pathogenic to papaya and *Vasconcellea monoica* (babaco‐mirim) (Bracale et al., 2020). *S. citrulli* (RG I isolates) was highly virulent on inoculated cantaloupe (*C. melo*) seedlings with a mean disease severity of 71%, while *S. cucurbitacearum* (RG II isolates) and *S. caricae* (RG IV isolates) were slightly virulent with a mean disease severity of 4%. *Phoma* (RG III isolates) had 0% mean disease severity and was nonpathogenic (Somai et al., 2002). However, Stewart et al. (2015) reported no differences in aggressiveness of the species across three cucurbit hosts *C. lanatus*, *Cucurbita moschata*, and *Cucumis sativus*. Differences in inoculum preparation may partially account for the different results. Somai et al. (2002) used a dilute sucrose‐casein solution to prepare conidial inoculum, whereas Stewart et al. (2015) used water. Previous studies have demonstrated that virulence of conidia of *Stagonosporopsis* spp. is dependent on an exogenous source of nutrients (Bergstrom et al., 1982; Svedelius & Unestam, 1978).

More recently, the genomes of *S. cucurbitacearum* strains Zq‐1 (Zhao et al., 2020) and DBTL‐4 (Wang et al., 2021) were analysed. After high‐throughput genome sequencing, assembly, and filtering out any low‐quality reads for *S. cucurbitacearum* strain Zq‐1, a 35.28‐Mb genome sequence was obtained. The genome sequence contained 9844 predicted genes, including 2066 genes that encode transmembrane proteins, 1024 genes that encode signal peptides proteins, 756 genes that encode secretory proteins, and 237 noncoding RNAs. In total, 605 proteins were identified from the Carbohydrate‐Active EnZymes (CAZyme) database, 130 proteins were identified from the Transporter Classification Database (TCDB), and 2869 proteins were identified from the Pathogen–Host Interactions (PHI) database. Additionally, 97 differentially expressed genes (DEGs) were matched in the PHI database while 36 DEGs were matched in the CAZyme database. Moreover, contig00011.93, an up‐regulated DEG, was reported to be involved in ATP‐binding cassette metabolism in infected leaves (Zhao et al., 2020).

The genome of *S. cucurbitacearum* strain DBTL4, made available by Wang et al. (2021), harbours 10,748 predicted protein‐coding genes, of which 812 encode secreted proteins. In the secretome, 113 proteins were reported as potential effectors that play important roles in deactivating host defence. Moreover, 635 CAZymes, 2273 transmembrane proteins, 143 transport proteins, and 3101 PHI proteins were identified. Comparing the assembly of strain DBTL4 (Zhao et al., 2020) with that of strain Zq‐1 revealed that the assembly of Zq‐1 in terms of number of scaffolds and scaffold N_50_ was better (Wang et al., 2021).

## 4 DISEASE SYMPTOMS AND LIFE CYCLE

**FIGURE 1:**
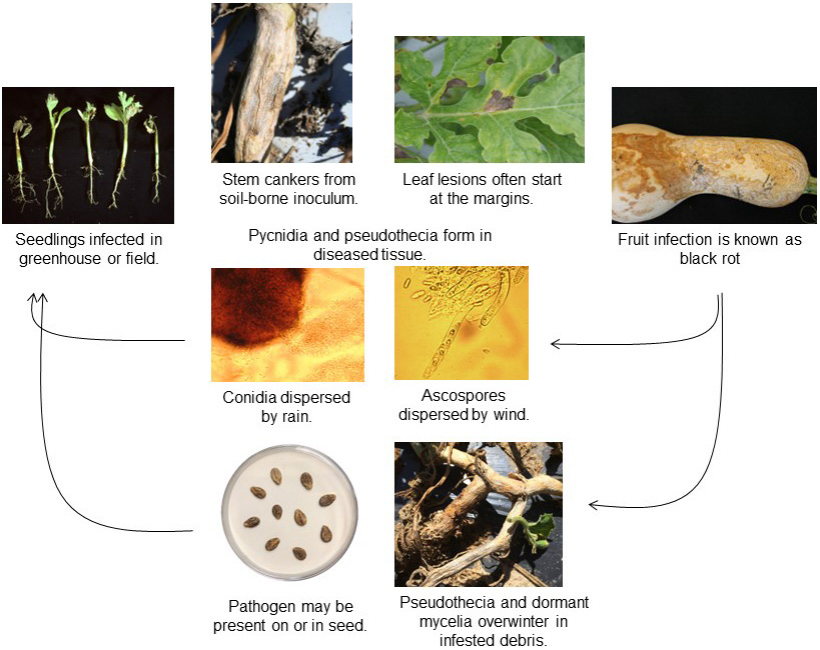
Disease cycle of gummy stem blight of cucurbit crops. Gummy stem blight pathogens can be seedborne or soilborne, overwintering as pseudothecia and dormant mycelium in infested debris. Under favourable conditions, pycnidia and/or pseudothecia form on diseased tissues and mature to release spores (rain‐splashed conidia or wind‐dispersed ascospores). Early symptoms include leaf lesions, which often start at the margins and can lead to stem cankers or fruit infection. Symptoms on infected fruit are known as black rot.

GSB can affect all aboveground vegetative and reproductive parts of cucurbits at any growth stage of the plant (Chester, 1891; Keinath, 2013). However, the degree of susceptibility varies among different cucurbits and species. Symptoms can appear 3–12 days after spore germination (Keinath, 2013). Leaf spots are one of the earlier observed symptoms on leaves or stems, especially on those that are shaded or that accumulate moisture for extended periods of time (Figure 1). The spots often start at the leaf margin or extend to the margins. Later, spots expand and coalesce, resulting in leaf blighting. The active lesions are typically water‐soaked and are visible on the underside of leaf and stem. The presence of water‐soaked lesions is due to the cell wall‐degrading enzyme polygalacturonase (PG), produced by *S. citrulli* (Zhang et al., 1999). A prominent PG isozyme produced by *Phomopsis cucurbitae* in decayed fruit was purified. The isozyme effectively macerated mature fruit tissue, suggesting it may be involved in the pathogenesis of *P. cucurbitae* (Zhang et al., 1999).

On the stem, lesions are circular and tan to dark brown. Cankers are observed on crowns, main stems, and vines (Choi et al., 2010; Rennberger & Keinath, 2018). Red to amber gummy exudates are often observed on the stems after cankers develop on cortical tissue (Figure 1).

GSB pathogens can cause a variety of symptoms on cucurbits. On fruit, GSB is called black rot. Fruit infection can be internal or external. Internal fruit rot always begins at the blossom end of the fruit. Internally the fruit shows brown discolouration that later progresses as it spreads to the outer surface of the fruit tip. Fruiting bodies appear as infection progresses, leading to shrivelled and blackened tissue (McPherson et al., 2011). Although some fruits exhibit external symptoms such as deformed tapering ends, internally infected fruits may not display obvious symptoms. Melon fruits inoculated with colonized potato dextrose agar disks displayed lesions, and 10‐day‐old fruits were more susceptible than older fruits (Zhang et al., 1999). The incidence of fruit rot tends to vary among seasons (Van Steekelenburg, 1984, 1985). Van Steekelenburg (1984) reported an infection percentage of 46% internally infected fruits. Externally, symptoms may vary with the host species. On cucumber, black rot usually develops during harvest or storage. The fruit develops small water‐soaked spots with gummy exudates. They appear with soft, black‐green lesions with shrunken tissue on the surface. The fruit shape has a subtle tapering on the flower end. On watermelon, fruit lesions appear as distinct, circular, greenish tan to black expanding pits that typically develop on the blossom end of the fruit. Under favourable conditions, pycnidia and pseudothecia develop near black centres of lesions. The presence of fruiting bodies helps distinguish between black rot and blossom end rot. Gummy exudates do not appear on decayed watermelon fruit. On winter squash, the fruit symptoms start before harvest. On butternut squash, orange to white fossilized areas develop as concentric circles where pycnidia are enclosed in the fruit tissue.

The optimum conditions that favour GSB development are warm weather (around 24–25°C) and high relative humidity (85%) and moisture (1–10 h of persistent moisture) (Svedelius & Unestam, 1978; Van Steekelenburg, 1985). The presence of free water and open wounds increase GSB symptom development on the leaves and stems (Svedelius & Unestam, 1978). Free water for at least 1 h is necessary for infection.

GSB causal agents are facultative necrotrophs that produce enzymes that aid in GSB development (Svedelius, 1990; Blakeman, 1971). They produce pycnidia (asexual fruiting bodies) and pseudothecia (sexual fruiting bodies). Pycnidia form earlier than pseudothecia, although some reports indicate that pseudothecia can form earlier (de Neergaard, 1989). The mean number of fruiting bodies/cm^2^ of leaf area ranges from 222 ± 58 to 579 ± 58 (Keinath, 2014). Young pycnidia are tan in colour and have a dark ring of cells around the ostiole (Keinath, 2013). Conidia, which are 6–13 μm long, are hyaline, cylindrical with round ends, and non‐ or monoseptate and serve in short‐distance dispersal. Pseudothecia are dark in colour and subglobose to flattened and have a diameter of 125–213 μm with a slight thickening in the basal–lateral wall (Corlett, 1981) (Figure 1). Ascospores, which are 14–18 × 4–6 μm, faintly pluriguttulate, hyaline with round ends, and monoseptate with a constriction at the septum, are the primary source of inoculum and can be dispersed over long distances (Rennberger et al., 2021). Eight ascospores per ascus are found. Ascospores are dispersed by rain and during night times with dew periods (Schenck, 1968). Spore germination is encouraged by the emission of volatile compounds from the leaf surface and exogenous nutrients (Pharis et al., 1982; Svedelius & Unestam, 1978). The pathogen can overwinter in crop debris for more than 2 years (Keinath, 2008) and can survive in planta as dormant mycelium (Chiu & Walker, 1949) or on seeds and cucurbit weeds (Figure 1). Mechanical injury, feeding of insects, and other infectious diseases such as powdery mildew may predispose the plant or create entry sites, thus promoting GSB development (Bergstrom et al., 1982). However, in another study, Rennberger et al. (2019) found a positive association between GSB and powdery mildew on watermelon leaves in only one of four seasons.

## 5 RESEARCH TOOLS

Young GSB lesions can be difficult to visually distinguish from lesions of anthracnose and target leaf spot. Symptoms of Phomopsis black root rot, caused by *P. cucurbitae*, and botrytis rot, caused by *Botrytis cinerea*, can be mistaken for GSB symptoms on greenhouse cucumber. Cultures of *Stagonosporopsis* should undergo standardized techniques and all features should be inspected to avoid any misidentification (Domsch et al., 1980). Pure cultures can be obtained from leaf or petiole lesions. Dried leaves must be rehydrated to initiate isolation. Small pieces (3 × 3 mm) are cut from the margin, surface‐disinfested with 0.6% sodium hypochlorite for 1 min, and then rinsed with sterile water. The dried pieces are then grown on ¼‐strength potato dextrose agar amended with antibiotics. To induce pycnidia and conidia production in 3 days, a photoperiod of 16 h is required (Keinath, 2013). A pure culture from one conidium can be obtained by immersing a 3 × 3‐mm piece from a colony with pycnidia into 2–4 mL sterile water and shaking for 20–30 s. Spore suspension (0.1 mL) is transferred onto water agar amended with antibiotics. Conidia will start germinating in as little as 20–24 h. Under a stereomicroscope at 30× magnification, an individual conidium and its germ tube can be excised and placed on ¼‐strength potato dextrose agar.

The most widely used test for detection in seeds has been the blotter test (Tian et al., 2017). According to the method approved by the U.S. National Seed Health System (https://seedhealth.org/files/2018/04/Cb‐2.2‐Didymella‐bryoniae‐blotter.pdf), 1000 seeds are surface sterilized, dipped in Dicloran, and incubated in the dark for 10 days at 25–27°C. Any seedlings with symptoms are then incubated further under a 12/12 h light/dark photoperiod for 4–5 days to induce the formation of pycnidia. Seedlings are observed under a stereomicroscope to record the presence of pycnidia and pseudothecia. Spore morphology and size are used to confirm the identity of the causal agent as one of the three GSB causal agents, but the three *Stagonosporopsis* species are morphologically indistinguishable (Stewart et al., 2015). Further identification at the species level relies on molecular methods.

Detection and identification methods that rely on microscopy (Keinath et al., 1995; Lee et al., 1984) are still being used to recognize *Stagonosporopsis* species but are more time consuming, less reliable, and less sensitive than molecular methods (Ling et al., 2010), and are not suitable for distinguishing among species of GSB pathogens. Molecular techniques are rapid, more sensitive, specific, and efficient (Keramas et al., 2004). Molecular tools for the detection and identification of GSB causal agents have been studied since the 1990s (Yao et al., 2016).

An early molecular diagnostic test was developed to identify GSB pathogens based on RAPD analysis (Keinath et al., 1995). The generated RAPD amplification patterns with oligonucleotide primers facilitated distinguishing between the GSB pathogen and *Phoma* species that can occur on cucurbit seedlings. To generate the fingerprint, the assay requires many short random primers. The primers require low annealing temperatures, which translates into variation in the band pattern and a possibility of incorrect diagnosis. For example, three isolates identified as belonging to RG I were shown to be *S. cucurbitacearum*, and one isolate identified as RG I was *S. caricae* (Kothera et al., 2003; Somai et al., 2002; Stewart et al., 2015).

Somai et al. (2002) developed a PCR‐ELISA for the detection and differentiation of *D. bryoniae* from *Phoma* species with similar morphology. Specific PCR primers were developed and modified by attachment of biotin and fluorescein. After amplification, the products were tested using ELISA. The assay was successful in detecting *D*. *bryoniae* and *Phoma* sp. However, the high background signal in PCR‐ELISAs limits their practical value in general disease diagnosis.

To facilitate species identification, Brewer et al. (2015) developed species‐specific PCR primers for a multiplex conventional PCR assay where primer Db01 amplifies a 356–374‐bp fragment of only *S. citrulli* isolates, Db06 amplifies a 283–289‐bp fragment in *S. citrulli* or a 286‐bp fragment of *S. cucurbitacearum* isolates, and Db05 amplifies a 216–224‐bp fragment in all three species.

These assays have been able to detect and identify the GSB causal agent from pure cultures and infected plant tissue. However, the small pathogen population and the presence of inhibitory compounds in seeds limit the application of PCR for seed health testing (De Boer et al., 1995). For improved sensitivity in seed detection, an assay that uses a combination of magnetic capture hybridization and multiplex real‐time PCR was developed (Ha et al., 2009). Single‐stranded DNA hybridization capture probes were covalently attached to magnetic particles and used to specifically concentrate the DNA template from cucurbit seeds. The obtained DNA was later amplified using a pathogen‐specific TaqMan PCR assay. The technique was successful in detecting the presence of *S. citrulli* in both watermelon and melon seeds and was 10‐fold more sensitive than direct real‐time PCR. However, the assay was only able to detect isolates of *S. citrulli* (referred to as RG I) and not all genotypes (Ha et al., 2009; Somai et al., 2002).

A real‐time PCR system for the detection of *S. citrulli* and *S. cucurbitacearum* (RG I and RG II) genotypes was developed (Ling et al., 2010). The assay can consistently detect these two GSB causal agents. The National Seed Health System has approved a modified version of this assay on a seed sample size of 10,000–30,000 seeds (https://seedhealth.org/files/2018/04/Cb‐2.1‐Didymella‐bryoniae‐PCR.pdf).

The primer set developed by Ling et al. (2010) was not used in a direct seedling health assay. Yao et al. (2016) developed a rapid, sensitive, and visual loop‐mediated isothermal amplification (LAMP) assay for the detection of GSB in seedlings. The assay had 1000‐fold higher sensitivity than conventional PCR and was completed in 45 min. Additionally, it could detect GSB in young muskmelon. The LAMP assay can be employed in early disease detection and may eventually reduce the risk of epidemics.

To identify which species were responsible for GSB outbreaks occurring since 2008 in Karnataka, India, Garampalli et al. (2016) used the ITS ribosomal DNA and a PCR‐based marker previously developed by Brewer et al. (2015) to identify seven isolates as *S. caricae* and two as *S. citrulli*. Additionally, Nuangmek et al. (2018) successfully identified the causal agent of GSB on cantaloupe in Thailand by conducting a phylogenetic analysis on combined sequences of the ITS and large subunit regions of ribosomal DNA, and β‐tubulin genes. The causal agent was identified as *S. cucurbitacearum*.

Long‐term storage of these pathogens is achieved through dried cultures on sterile filter paper (Keinath, 2013). A circular 75‐mm sterile filter paper is centred on a 100‐mm Petri dish with ¼‐strength potato dextrose agar. Two pieces of a pre‐established culture are placed on the edge of the agar and not the filter paper. After the colony covers the filter paper, the filter paper is removed with sterile forceps and then stored for 3–5 days under a laminar flow hood. Pieces of the dry filter paper (0.5–1 cm^2^) are stored in sterile vials at 5°C. These cultures can remain viable for several years. Reviving these cultures is done by placing one or two pieces of the filter paper onto potato dextrose agar, followed by incubation at 20–25°C with a photoperiod of 16 h.

## 6 DISEASE MANAGEMENT

Management of GSB involves integrating cultural and chemical practices. The use of certified disease‐free seeds (Keinath, 1996a, 1996b), seed treatment (Hopkins et al., 2003), removal of weeds and volunteer plants, cultural practices that reduce residue (Keinath, 2002), and crop rotation with nonhost crops (Keinath, 1996a, 1996b) can help in GSB management. Genetic resistance sources against GSB have been discovered (Gusmini et al., 2005); nonetheless, no GSB‐resistant cultivars are currently commercially available. Growers continue to rely heavily on fungicides in managing GSB; however, GSB causal agents have developed resistance to several of the fungicides developed for their control (Thomas et al., 2012).

Plant disease management starts with prevention. Several cultural practices that reduce or eradicate GSB inoculum before a cucurbit crop is planted delay or slow epidemics of GSB. The use of certified disease‐free seeds is crucial in preventing the establishment of GSB in greenhouse and field plantings.

Sowing seed infected even with low levels of seedborne inoculum can result in high levels of disease in greenhouse and field plantings (Keinath, 1996a, 1996b; Neergard, 1977). Sudisha et al. (2006) demonstrated that GSB can be transmitted from seed to plant and vice versa. Moreover, spread of GSB in greenhouses via transplants has been reported where three glasshouses obtained seeds from one source (Brown et al., 1970). A common pattern in a greenhouse flat is a dead plant with GSB from a contaminated seed that is surrounded by plants with symptoms. In one study, 11%–15% of the seedlings adjacent to the dead plant showed GSB symptoms (Keinath, 2016).

Seed treatment is necessary to reduce inoculum linked with seeds. Mancozeb seed treatment reduced disease incidence from 39% in untreated seeds to 13% or less (Sudisha et al., 2006). Additionally, seed treatment with 1600 μg/mL of peroxyacetic acid for 30 min followed by seed drying at low humidity in a 40°C drying oven for 48 h was also found effective in preventing seed transmission of GSB (Hopkins et al., 2003).

Volunteer cucurbit seedlings, which emerge from seed that remain in soil when fruits are left in the field after harvest, can also serve as a source of inoculum and must be eradicated prior to planting. For example, the incidence of watermelon volunteers with GSB symptoms increased from 11% to 66% in a 2‐week period (author's unpublished data). Weeds in the cucurbit family can serve as hosts for *Stagonosporopsis* spp. Balsam pear (*Momordica balsamina*), buffalo gourd (*Cucurbita foetidissima*), citron melon (*Citrullus caffer*), colocynth (*Citrullus colocynthis*), finger leaf gourd (*Cucurbita digitata*), ivy gourd (*Coccinia grandis*), smell melon (*Cucurbita foetidissima*), and white bryony (*Bryonia alba*) have been reported as hosts for *S. cucurbitacearum* and *S. citrulli* (Farr & Rossman, 2022; Levi et al., 2001; McCreight, 2017; Rennberger & Keinath, 2018).

Due to the ability of the GSB causal agents to survive on crop residue, cultural practices that reduce residue help in disease management (Keinath, 2002, 2008). Deep turning of infected debris from the previous season was found to reduce the primary inoculum (Keinath et al., 1999). Keinath (2002) reported that the GSB pathogens on buried debris will still produce conidia. However, conidia produced on watermelon tissue buried for more than 16 weeks did not infect watermelon seedlings. An effective yet simple way to reduce the survival of the GSB pathogens is by removing the polyethylene mulch and incorporating the debris completely into the soil through tillage (Keinath, 2008). This technique can reduce inoculum, making it possible to plant susceptible cucurbits every other year. After harvest, in muskmelon fields abandoned for up to 6 months until the field is prepared for the winter, a 3‐year rotation is required to eliminate inoculum from debris (Keinath, 2008).

A 1‐ to 3‐year rotation with nonhost crops is highly recommended to reduce GSB incidence. A 1‐year rotation of spring wheat followed by summer soybean has been reported to successfully reduce disease severity on watermelon planted in the following season (Keinath, 1996a, 1996b). Additionally, the use of cabbage residue followed by soil solarization has been effective in increasing the size and number of healthy marketable fruit when compared with nonsolarized treatments (Keinath, 1996a, 1996b).

GSB is currently found on 37 species of cucurbits from 21 different genera (Keinath, 2011; Rennberger & Keinath, 2018). Nevertheless, susceptibility varies among these cucurbits. *C. melo* and *C. lanatus* are the most susceptible species (Keinath, 2014), while *Cucurbita* spp. have consistently been reported as less susceptible (Chiu & Walker, 1949; dos Santos et al., 2009; Keinath, 2014).

Sources of genetic resistance to GSB continue to be studied. Screening for resistance has been performed on the major cucurbit crop species using a variety of traditional and molecular‐assisted breeding methods (Boylan et al., 1994; Gusmini et al., 2017; Han, Dong, Liu, et al., 2022; Han, Dong, Shi, et al., 2022; Hong et al., 2022; Luo et al., 2022; Ma et al., 2022; Wehner & Shetty, 2000; Wehner & St Amand, 1993; Zhang et al., 1995; Zhang et al., 1997).

In early studies with watermelon, cv. Congo was found least susceptible, whereas Fairfax was of intermediate susceptibility (Schenck, 1962). Plant Introduction (PI) 189225 and PI 271778 were identified as the most resistant in the USDA‐ARS watermelon germplasm collection at the time (Sowell, 1975). Norton (1979) initially reported that in PI 189225 resistance was mediated by one gene. However, more recent studies indicate that several genes with minor effects are responsible for this trait (Gusmini et al., 2017; Hassan et al., 2019). Ren et al. (2020) found that in PI 189225, a region on chromosome 8 accounts for approximately 32% of the phenotypic variation in GSB resistance. Gimode et al. (2020) identified three GSB resistance quantitative trait loci (QTLs) (ClGSB3.1, ClGSB5.1, and ClGSB7.1) in an F_2:3_ interspecific *Citrullus* mapping population (*n* = 178). The cross was between *C. amarus* GSB‐resistant PI 482276 and *C. lanatus* ‘Crimson Sweet’. Several potential candidate genes all associated with plant defence against pathogens were identified in the study, including gene ClCG07G013230 in ClGSB7.1, which encodes an Avr9/Cf‐9 rapidly elicited protein 146 homologue with a mutation in the DUF761 domain that is highly linked to GSB resistance (Hu et al., 2017). Four single nucleotide polymorphisms (SNPs) were identified in gene ClCG07G013230, all leading to amino acid changes. A SNP in the DUF761 domain that was previously associated with disease resistance (Zhang et al., 2019) showed significant association with GSB resistance in the mapping population (Gimode et al., 2020).

In melon (*C. melo*), all available resistant cultivars are derived from PI 140471 (McGrath et al., 1993). With this source failing to achieve acceptable resistance (Sitterly & Keinath, 1996), new resistant lines from various cultigens of melon are being studied (Hassan et al., 2018). In 2009, AFLP markers linked to GSB resistance in PI 420145 were identified (CMCT505, CMTC160a+b220, ISSR‐5760, ISSR‐100900, and CMTA1701a) (Joseph, 2009). The AFLP markers were found to be directly linked to GSB resistance genes *Gsb‐1*, *Gsb‐2*, *Gsb‐3*, and *Gsb‐4* (Joseph, 2009). Recently, eight new genes conferring resistance to GSB have been identified on chromosome 4 (Hu et al., 2017), while a major QTL associated with resistance was identified on chromosome 1 (Hong et al., 2022). Even more recently, the screening of 260 melon germplasm resources in China resulted in the identification of a single dominant gene on chromosome 7 (Ma et al., 2022).

The US National Cucumber Germplasm Collection was screened for resistance to GSB. The most resistant were cultigens Homegreen, Little John, and Transamerica (Wehner & Shetty, 2000). In 2017, the first report of molecular markers and genetic mapping resistance to GSB in cucumber was published. A set of recombinant inbred lines (RILs) was used to detect QTLs that confer resistance. In PI 183967 two pairs of major QTLs and several minor QTLs were identified. Additionally, seven genes were found to be related to GSB resistance (Liu et al., 2017). In 2022, Han, Dong, Liu, et al. (2022) and Han, Dong, Shi, et al. (2022) reported major QTLs for GSB resistance on chromosomes 3 and 6.

Several sources for genetic resistance against GSB have been discovered (Gimode et al., 2020; Gusmini et al., 2005). However, to date no commercial cultivars with genetic resistance to GSB in the field have been released. A major limitation of many of the studies, even very recent ones, is the use of inoculation using only a single fungal strain identified as *D. bryoniae*, leaving uncertainty about whether the resistance identified will be effective against all three species of GSB pathogens. For example, the single isolate DB‐H‐23, used by St Amand and Wehner (1995a) to inoculate cucumber cultigens, is an isolate of *Phoma*, renamed NC2 by Somai et al. (2002), not an isolate of *Stagonosporopsis*. Among the eight isolates used in another study by St Amand and Wehner (1995b), DB‐C‐AZ is *S. cucurbitacearum* (isolate AZ1 in Somai et al., 2002), DB‐H‐23 was again represented, and three isolates (ATCC 56275, ATCC 36934, and DB‐WI) are *S. citrulli* (Somai et al., 2002; Stewart et al., 2015). Several disease resistance studies reported in 2022 employed single strains identified as *D. bryoniae*.

Using GSB‐resistant cultivars can help decrease growers’ reliance on fungicides, which can be expensive and are often associated with negative effects on human and bee health (Jones et al., 2020). Moreover, they may reduce yield loss associated with GSB. Because of the lack of commercially available resistant cultivars, fungicide application is considered the most effective method for GSB control. Several contact and systemic fungicides with varying modes of action are registered for use against GSB. Chlorothalonil and mancozeb, which belong to the chloronitrile and ethylene‐*bis*‐dithiocarbamate (EDBDCs) groups, respectively, are the most popular contact fungicides. In 2018, 53% of the watermelon acreage was treated with mancozeb (USDA‐NASS, 2021).

To reduce the spread of GSB under favourable environmental conditions, systemic fungicides are necessary in disease management to suppress sporulation of the GSB fungus after infection (Keinath, 2021). In a study on premixed fungicides to control GSB, cyprodinil plus fludioxonil and cyprodinil plus difenoconazole were the most effective in reducing the severity of GSB caused by *S. citrulli*, as well as leaf lesion size and the percentage of leaf lesions with fruiting bodies. Moreover, these fungicide combinations were found to be more effective than chlorothalonil alone or fludioxonil alone in reducing disease severity and the area under the disease progress curve (Keinath, 2021).

The GSB causal agents have developed resistance to several of the fungicides developed for their control (Table 1). Several registered fungicides for GSB are in Fungicide Resistance Action Committee (FRAC) groups 1, 3, 7, and 11. Resistance has been observed in benomyl and thiophanate‐methyl throughout the United States (Keinath & Zitter, 1998; Thomas et al., 2012). Additionally, the intensive use of azoxystrobin has led to resistance (Stevenson et al., 2008; Thomas et al., 2012). Isolates that previously developed resistance to azoxystrobin also demonstrated resistance to Pristine, a mixture of pyraclostrobin and boscalid (Avenot & Michailides, 2007; Keinath, 2015; Stevenson et al., 2008).

**TABLE 1:** Reports of fungicide resistance in *Stagonosporopsis* species causing gummy stem blight.

Fungicide class (FRAC)	Fungicide	Basis of resistance	References
Quinone‐outside inhibitors (11)	Azoxystrobin	Nucleotide substitution: glycine to alanine at amino acid 143 (G143A) in Cyt *b* of quinone‐outside inhibitor‐resistant *S. citrulli*	Finger et al. (2014)
	Pyraclostrobin	Limited to few isolates. Uncertain	Keinath (2015)
Succinate dehydrogenase inhibitors (7)	Boscalid	Nucleotide polymorphisms in codon 277 of the gene encoding SDH subunit B: replacement of a histidine residue with tyrosine (H277Y) or arginine (H277R) in resistant isolates of *S. citrulli*	Avenot et al. (2012)
	Fluopyram	I229V mutation in SdhB in fluopyram‐resistant *S. citrulli*	Li et al. (2019)
	Pydiflumetofen	Not determined	Mao et al. (2020
Demethylation inhibitors (3)	Tebuconazole	Uncertain	Li et al. (2019)
Methyl benzimidazole carbamates (1)	Thiophanate‐methyl	Uncertain	Thomas et al. (2014)

Resistance to the demethylation inhibitor fungicide tebuconazole, which was labelled in 2008, has been reported (Li et al., 2016), but this has been less frequent compared with other classes of fungicides (Keinath & Hansen, 2013).

Li et al. (2019) studied the fungicide resistance profiles of 113 *S. citrulli* and 19 *S. caricae* isolates using in vitro mycelial growth assays and molecular markers based on genes encoding fungicide targets. All the *S. caricae* isolates were resistant to azoxystrobin and tebuconazole, while *S. citrulli* isolates were all sensitive to tebuconazole and resistant to azoxystrobin except for two isolates. All *S. caricae* isolates were reported to be sensitive to the succinate dehydrogenase inhibitor (SDHI) fungicides boscalid and fluopyram, while *S. citrulli* isolates varied in their sensitivity to boscalid and all were sensitive to fluopyram except for one isolate. The fluopyram‐insensitive *S. citrulli* isolate appears to have a distinctive I229V mutation in succinate dehydrogenase subunit B at a target for SDHI fungicides. Phenotypic resistance to boscalid was observed in *S. citrulli*; however, these phenotypes were not linked with multilocus genotypes identified with 16 microsatellite loci. Moreover, isolates with the same multilocus genotypes varied by succinate dehydrogenase subunit B genotype.

Pydiflumetofen, an SDHI fungicide, is registered for the control of Sclerotinia rot and Fusarium head blight in rapeseed and wheat. Mao et al. (2020) tested the baseline sensitivity of 69 field isolates of the GSB pathogen for pydiflumetofen in vitro by evaluating the EC_50_ values. All field isolates showed strong hyphal growth inhibition, deeming pydiflumetofen as a potential fungicide to control GSB. Furthermore, pydiflumetofen‐resistant mutants MKR‐14, MKR‐15, MKR‐91, BB15R‐1, and BB15R‐3 were sequenced. A CAC‐to‐TAC mutation in the *SdhB* gene at codon 227 resulted in a substitution of histidine with tyrosine, suggesting that mutations in the *SdhB* gene can result in resistance of the GSB pathogen to pydiflumetofen (Mao et al., 2020).

Management of fungicide resistance is based on the assumption that the resistance tends to develop from repeated exposure to a high‐risk fungicide. Nevertheless, there have been reports of resistance in fields where the fungicide was never used. This reveals that resistant isolates might have been introduced from an outside source. Thomas et al. (2014) evaluated watermelon seeds as a potential source. Seeds (5800) obtained from commercial seed companies and commercial growers were tested for GSB fungi. The three isolates obtained were screened for sensitivity to four fungicides regularly used against GSB, (boscalid, tebuconazole, azoxystrobin, and thiophanate‐methyl). All three seedborne isolates were resistant to thiophanate‐methyl.

Alternative biopesticides have been considered in managing GSB. Currently, only one strain of *Bacillus subtilis* (QST 713, Serenade ASO) is available in the market for control of GSB. *B. subtilis* QST 713, root extract of *Reynoutria sachalinensis* (Regalia), hydrogen dioxide (Oxidate), paraffinic oil (JMS Stylet Oil), polyoxin D (Oso), and 92% edible fish oil + 5% sesame oil (Organocide) were evaluated for their protective effects against GSB on muskmelon seedlings (Keinath, 2016). Polyoxin D alone, *B. subtilis* QST 713 followed by Polyoxin D, and *R. sachalinensis* extract followed by *B. subtilis* QST 713 were as effective as mancozeb in reducing the severity of GSB (Keinath, 2016). In the field, Polyoxin D was as effective as chlorothalonil and more effective than *R. sachalinensis* extract, which did not significantly reduce GSB on cantaloupe, honeydew, or watermelon (Jones et al., 2020). Tank‐mixing or alternating these two biofungicides with several conventional fungicides in FRAC groups 3, 7, and 9 was also as effective as chlorothalonil treatment.

The effects of chemical and biological treatments on GSB were evaluated by Utkhede and Koch (2002). Out of the four experiments conducted, myclobutanil, kresoxim‐methyl, and azoxystrobin were able to control GSB in three of the experiments, while *Enterobacter agglomerans* (B8Fr), *B. subtilis* (AGS‐4), and lysozyme applied as sprays on lesions controlled GSB in one experiment.

To further incorporate organic methods in GSB control, nitrogen fertilizers were replaced with chicken manure. *Bacillus polymyxa* was applied on the seed and to the foliage. Disease incidence decreased by 68% in 2000 and 52.9% in 2001. Moreover, there was an increase in vigour (up to 257%), yield (97% and 67%), and fruit number (up to 41%) (El Meleigi & Al‐Rehiayni, 2004). In treatments with *Aspergillus terreus* (ATKGM1) and *Penicillium purpurogenum‐2* (PPDMK‐2), in vitro inhibition of GSB pathogens was minimal, but in greenhouse trials, disease incidence was reduced by 43.8% by *A. terreus* (ATKGM1) and by 44.6% with *P. purpurogenum‐2* (PPDMK‐2) (Mangala & Rajkumar, 2018) compared to the negative control (96.5% disease incidence).

## 7 CONCLUSION AND FUTURE PROSPECTS

The causal agent of GSB is now recognized as a complex of three closely related but phylogenetically distinct species. The disease occurs in 37 species of cucurbits from 21 different genera in the Cucurbitaceae family. *S. cucurbitacearum* and *S. citrulli* are specific to cucurbits, whereas *S. caricae* is also pathogenic to at least two fruits in the Caricaceae family. Under favourable environmental conditions, the pathogen can infect all aboveground parts of susceptible hosts. GSB can decrease yield and fruit quality by 17%–43%. Identifying the causal agent of GSB at the species level based on morphology is challenging; hence, species‐specific PCR primers based on microsatellite markers are used currently (Brewer et al., 2015).

Management of GSB heavily relies on fungicides. Thus, it is no surprise that *Stagonosporopsis* spp. developed resistance to several fungicides used for control of GSB. Introducing new fungicides can aid in managing GSB. Polyoxin D is a promising biopesticide. Moreover, to reduce the rapid development of resistance, management of GSB requires the integration of both cultural practices and rotations of fungicide active ingredients in different FRAC groups. Despite the identification of several new sources for genetic resistance against GSB, no resistant cultivars have been released. Introducing resistant varieties and new biopesticides to the market could help growers reduce their reliance on fungicides and hence delay or even prevent resistance to additional fungicides.
